# Patched Knockout Mouse Models of Basal Cell Carcinoma

**DOI:** 10.1155/2012/907543

**Published:** 2012-09-13

**Authors:** Frauke Nitzki, Marco Becker, Anke Frommhold, Walter Schulz-Schaeffer, Heidi Hahn

**Affiliations:** ^1^Department of Human Genetics, University Medical Center Göttingen, Heinrich-Düker Weg 12, 37073 Göttingen, Germany; ^2^Department of Neuropathology, University Medical Center Göttingen, Robert-Koch-Straße 40, 37075 Göttingen, Germany

## Abstract

Basal cell carcinoma (BCC) is the most common human tumor. Mutations in the hedgehog (HH) receptor Patched (PTCH) are the main cause of BCC. Due to their high and increasing incidence, BCC are becoming all the more important for the health care system. Adequate animal models are required for the improvement of current treatment strategies. A good model should reflect the situation in humans (i.e., BCC initiation due to *Ptch* mutations on an immunocompetent background) and should allow for (i) BCC induction at a defined time point, (ii) analysis of defined BCC stages, and (iii) induction of BCC in 100% of animals. In addition, it should be easy to handle. Here, we compare several currently existing conventional and conditional *Ptch* knockout mouse models for BCC and their potential use in preclinical research. In addition, we provide new data using conditional *Ptch* ^*flox*/*flox*^ mice and the *K*5-*Cre*-*ER* ^*T*+/−^ driver.

## 1. General Aspects and Current Therapies of BCC

### 1.1. Epidemiology

BCC is a tumor of the skin and the most prevalent cancer in the Western world. Its incidence is increasing worldwide. Retrospective studies show that the increase in mainland Europe is approximately 1/100,000 persons per year and even 6/100,000 in the UK [1]. It is estimated that the lifetime risk of developing BCC for a child born in 1994 is 28% to 33% [2] and that young people will suffer more and more from this tumor [3]. Risk factors for BCC formation are exposure to ultraviolet radiation (UV) or ionizing radiation (IR), immunosuppression, or a genetic predisposition [4]. Due to their high and increasing incidence, BCC are becoming an important issue for the health care system [5]. In some countries, the cost of care for BCC and other nonmelanoma skin cancers comprises 9% of the costs for all cancers [6].

### 1.2. Histology

BCC are usually well differentiated and the tumor cells appear histologically similar to basal cells of the epidermis. BCC can be subdivided into two subgroups that show either an indolent or an aggressive growth behavior. The indolent-growth variants comprise nodular/micronodular and superficial BCC. These subtypes occur in 21%/15% and 17% of cases, respectively, and thus are the most common BCC variants [7]. Whereas nodular BCC consist of nests of basaloid cells in the dermis, superficial BCC are characterized by numerous small tumor nests attached to the undersurface of the epidermis by a broad base. The more aggressive tumors are less frequent and include infiltrative, metatypical, morpheaform, or sclerosing BCC (for review see [8]). Although BCC very rarely metastasize, they can result in local tissue destruction due to invasion into deeper layers of the skin, thereby causing significant morbidity [9]. 

### 1.3. Molecular Pathogenesis of BCC

BCC are thought to be caused by uncontrolled activation of the hedgehog (HH) signaling pathway. In the majority of cases, this is due to inactivating mutations in the HH receptor and tumor suppressor gene *PTCH*. *PTCH* mutations in BCC were first observed in basal cell nevus syndrome (also known as nevoid basal cell carcinoma syndrome or Gorlin-Goltz syndrome), which is a rare familial autosomal-dominant disorder that predisposes the affected individual to developing this tumor. Only a minority of BCC are caused by activating mutations in Smoothened (SMO) (reviewed in [9]). 

PTCH normally acts as an inhibitor of HH signaling by repressing the function of SMO. Binding of the HH ligand to PTCH or inactivating *PTCH* mutations suspend this inhibition, which allows activation of SMO and results in the formation of activator forms of the GLI zinc finger transcription factors GLI2 and GLI3. Activation of GLI2 and GLI3 leads to transcription of *GLI1*. Thus, the expression level of *GLI1* is considered as a reliable indicator of the pathway's activity. Another HH target is *PTCH* itself, which regulates its expression in a negative feedback (reviewed in [10]). Indeed, nearly all BCC express *GLI1 *and* PTCH*, which demonstrates the important role of aberrant HH signaling in these tumors [11]. 

Several other signaling pathways are presumably involved in BCC tumorigenesis. Mutations of the tumor suppressor gene *p53* have been shown in 40% of sporadic BCC [12] and were correlated with aggressive behavior [13–15]. In addition, activation of canonical Wnt/*β*-catenin signaling seems to play a role in specific histological BCC subtypes. These subtypes include early stages of superficial BCC [16], pilomatricoma (a tumor of the hair follicle [17]) as well as infiltrative BCC variants [18, 19]. Indeed, nuclear *β*-catenin is found in infiltrative BCC and in superficial BCC [18], but only rarely in human nodular BCC [17, 18, 20]. BCC also express activated AKT [21]. Finally, EGFR signaling seems to be an essential *in vivo* requirement in HH-driven BCC because EGFR signaling cooperates with the HH pathway to induce genes (e.g., *JUN*, *SOX9,* and *FGF19*) critical for the determination of the oncogenic BCC phenotype [22]. 

Growth and progression of human BCC is also highly influenced by the tumor microenvironment. For example, tumor-associated macrophages are able to enhance the invasive phenotype and angiogenesis [23]. Furthermore, *α*-smooth muscle actin positivity of peritumoral fibroblast tends to be greatest in infiltrative tumor areas [24]. In addition, stromal cells of BCC produce high levels of *Gremlin1*, which is a factor stimulating BCC growth by antagonizing bone morphogenic protein-mediated repression of cell proliferation [25, 26]. Finally, EGFR ligands are increased in the tumor stroma [27], which may influence tumor intrinsic EGFR signaling (see above).

However, whereas all the above-mentioned factors may influence the susceptibility to BCC or the BCC phenotype, deregulation of HH signaling is the central abnormality in all these tumors and seems to play the major role in its formation [9].

### 1.4. Conventional Treatment Options of BCC

Surgical excision is currently by far the most commonly used treatment of BCC. However, surgery can result in permanent tissue damage and scarring, which is unwanted especially in facial areas. In addition, surgery may be problematic if the tumor is localized around the eye, mouth, or in close vicinity of the cartilage of the nose and ears [9]. This has led to less invasive treatment strategies such as photodynamic therapy or application of imiquimod-containing creams. Photodynamic therapy refers to a technique in which the tumor is treated with a photosensitizing chemical in a cream and is exposed to light several hours later [28]. Imiquimod is an immune response modifier, which stimulates the Toll-like receptor 7 and increases the activity of natural killer cell, macrophages, and the proliferation and differentiation of B lymphocytes [29]. Another option is cryotherapy, which destroys the skin lesion by application of extreme cold such as compressed nitrous oxide [30]. Another agent for topical application is 5-fluorouracil (5% cream), which leads to tumor necrosis. Among the drawbacks of the latter agent is the limited tissue penetration [31].

Although these therapies are associated with moderate morbidity, the outcome is still considered to be unspecific. In addition, these treatments sometimes have side effects such as pain, scarring, and local skin reactions [31]. Together, these data show that the availability of a simplified and more effective treatment would contribute to lower the costs related to this tumor. 

### 1.5. Targeting the HH Signaling Pathway in BCC

The knowledge about the genetic and molecular events involved in BCC pathogenesis has enormously contributed to the establishment of new treatment options. Very successful have been strategies specifically targeting HH signaling. The first small-molecule inhibitor of the HH pathway was the naturally occurring compound cyclopamine that inhibits SMO activity by direct binding [32]. Within the last few years, more potent SMO inhibitors have been developed and are currently being tested in phase I and II clinical trials [33]. Recently, the SMO antagonist vismodegib (Erivedge, GDC-0449) has been approved by the FDA for the treatment of metastasizing and locally destructive BCC [34, 35]. However, although vismodegib shows both remarkable therapeutic and preventive efficacy, the cumulative toxicity of this agent has led to discontinuation of therapy in a substantial fraction of patients [35, 36]. Therefore, it will be necessary to develop strategies that ameliorate some of the common toxicities of this drug [35].

## 2. Mouse Models of BCC for Preclinical Studies

The establishment of new treatment strategies requires adequate animal models. An ideal model should allow for analysis or modulation of molecular events associated with tumor initiation or tumor progression. It should also permit to evaluate antitumor therapies useful to prevent, inhibit, or even to induce regression of BCC *in vivo*. To fulfill these requirements, an ideal animal model should allow for analysis of BCC that have reached a defined BCC stage after their initiation in 100% of animals.

Hitherto, several murine BCC models exist. These include *Ptch* knockout mice and mice overexpressing Hh, oncogenic Smo, Gli1 or Gli2 specifically in the skin using the keratin (K) 5, 6, or 14 promoters. Depending on the gene and the targeted cell type, the skin tumor subtypes range from follicular hamartoma and trichoepithelioma to nodular or invasive BCC [16, 37–45]. In addition, allografts from BCC-bearing *Ptch* ^+/−^ *p*53^−/−^ mice or from Shh transgenics can be grown in scid mice [43, 46]. Finally, the cell line ASZ001 generated from a BCC of an irradiated *Ptch* heterozygous mouse (see below) has been successfully implanted into nude mice and used to study the effects of the EGFR-inhibitor afatinib [22]. 

Since most human BCC arise due to *PTCH* mutations and since the stromal microenvironment plays an important role in formation and progression of this tumor (see section “*Molecular pathogenesis of BCC”*), immunocompetent *Ptch* mutant mice certainly represent the closest model to the human condition. 

## 3. *Ptch* Knockout Mouse Models for BCC

### 3.1. Spontaneous *Ptch* Mutations in Mice

Two spontaneous *Ptch* mutant animals have been described. The spontaneous recessive mutation “mesenchymal dysplasia” (mes) is caused by a deletion of 32 bp in the C-terminal cytoplasmic domain of *Ptch* [47]. *Ptc*1^*mes*/*mes*^ mice are viable and show increased proliferation and hyperplasia of the basal cell layer [48]. However, in spite of these skin anomalies *Ptc*1^*mes*/*mes*^ mice do not develop BCC even after exposure to radiation [49]. 

The *Ptch1D11* is a mutation caused by an aberrant recombination event while producing a *Ptch* null allele for the generation of *Ptch* ^*neo*12/+^ mice (see below). The *Ptch*1^*D*11^ locus presumably results in a weak *Ptch* allele [50]. *Ptch*1^*D*11/*D*11^ animals are sterile, but otherwise appear normal [50]. 

### 3.2. Conventional *Ptch* Knockout Mice

So far, two different conventional *Ptch* knockout mouse models for BCC have been described. These are the *Ptch* ^*neo*12^ and *Ptch* ^*neo*67^ strains, in which exons 1 and 2 or exons 6 and 7, respectively, are deleted in the germline [51, 52]. Homozygous *Ptch* ^*neo*12/*neo*12^ and *Ptch* ^*neo*67/*neo*67^ embryos die around embryonic day 9.5 due to heart and neural tube closure defects. Heterozygous *Ptch* ^*neo*12/+^ and *Ptch* ^*neo*67/+^ animals survive and show increased susceptibility to spontaneous formation of rhabdomyosarcoma, medulloblastoma, and tiny epidermal hyperproliferations. To induce BCC, *Ptch* ^*neo*12/+^ animals are usually exposed to UV three times per week for up to several months [53–55]. After 12 months of chronic UV exposure, all *Ptch* ^*neo*12/+^ mice develop lesions with histologic features of human BCC. Of these lesions, 44% can be classified as superficial, 13% have histologic features of nodular or infiltrating human BCC and 43% have features of trichoblastoma [53]. Chronic UV exposure also results in macroscopic tumors. Of these visible tumors, approximately 20% are BCC or trichoblastomas (tumors with follicular differentiation that share many histologic features with BCC), 30% are squamous cell carcinoma (SCC) or keratoacanthomas (SCC-like tumors), and 50% are fibrosarcomas or fibromas [53]. 

The situation is somewhat different when inducing BCC-like lesions by IR. As shown by Aszterbaum et al. [53], a single dose of 1–4 Gy applied at 2 months of age results in microscopic trichoblastoma-like tumors in all *Ptch* ^*neo*12/+^ mice after 1 year. Another study performed by Mancuso et al. revealed that a dose of 3-4 Gy applied to adult *Ptch* ^*neo*67/+^ mice at the age of 2-3 months leads to nodular BCC-like lesions in 21–47% of animals, and in infiltrative lesions in 5–12% [45]. IR exposure never results in fibrosarcomas or SCC [45, 53]. Particularly BCC in the IR-induced model further progress into an aggressive phenotype [45]. Immune surveillance was not impaired in either model [53]. 

On the molecular level, formation of IR-induced nodular BCC requires *Ptch* heterozygosity in conjunction with mutations in other molecules such as *p53* [45]. Moreover, the progression into an aggressive phenotype seems to be associated with biallelic loss of *Ptch* [45]. This might be different from human BCC, which in most cases lack aggressiveness [56] and which frequently show loss of heterozygosity at the *PTCH* locus on chromosome 9q22 already at the nonaggressive stage [57–59]. Thus, it remains to be resolved whether loss of the wildtype* Ptch* allele in irradiated mice indeed triggers BCC aggressiveness or whether it is just a secondary event due to general irradiation-induced genomic instability. 

These differences to human BCC and the fact that BCC in irradiated *Ptch* heterozygous mice develop at undefined time points and in indefinite areas of the exposed skin render this animal model may complicate the examination of early molecular processes involved in the initiation of BCC. However, these mice are a great tool to evaluate new treatment options of microscopic, macroscopic, and aggressive BCC that are caused by *Ptch* mutations along with additional irradiation-dependent mutations. Indeed, irradiated *Ptch* ^*neo*12/+^ knockouts have been used in several preclinical studies (Table 1), which are described in the following section. 

To study the effects of the Hh inhibitor cyclopamine [60], BCC have been induced in *Ptch* ^*neo*12/+^ animals by UV exposure 3 times per week from age 6 to 32 weeks. After this time, approximately 50% of the mice had developed one or more macroscopic BCC. For the following 20 weeks the animals were treated with cyclopamine that significantly reduces tumor growth [60]. Regression of microscopic BCC after Hh inhibition has also been shown in skin punches of UV-irradiated *Ptch* ^*neo*12/+^ mice, which were kept in cell culture for 6 days and treated for the last 4 days with the small molecule inhibitor of Hh signaling CUR61414 [61]. 

*Pt* *ch* ^*neo*12/+^ animals have also been used to analyze the antitumoral effects of *α*-difluoromethylornithine (DFMO) [62]. DFMO is a potent inhibitor of cutaneous ornithine decarboxylase, which is expressed in BCC and is known to promote tumor formation [69, 70]. To analyze its antitumoral effects, *Ptch* ^*neo*12/+^ animals were irradiated with UV 3 times per week for 32 weeks [62]. Thereafter, the tumor-bearing animals obtained DFMO in the drinking water for 20 weeks. The results show that DMFO reduced the number of visible BCC and diminished BCC-like microscopic lesions. Furthermore, a reduction of *Ptch*, *Gli1*, *Gli2,* and *Gli3* expression in nontumor-bearing skin of these animals was evident [62]. 

A fourth study analyzed the antitumoral activity of the retinoid tazarotene [55]. Retinoids are ligands of the retinoic acid receptor (RAR) and the retinoid X receptor (RXR) and show tumor-suppressive capacity in several tumor entities [71]. Tazarotene was topically applied to the skin of 1.5 or 2.5 months old *Ptch* ^*neo*12/+^ mice for 5 consecutive days/week. Two weeks after onset of treatment, that is, at the age of 2 or 3 months, BCC were induced by exposure to UV (3 times/week) or IR (once), respectively. In order to examine the growth of microscopic BCC, skin biopsies of the UV-treated group were taken at the ages of 7, 9, and 11 months, whereas those of the IR-treated group were taken at the age of 10 months. Tazarotene treatment reduced the number and size of microscopic BCC after UV or IR exposure and also prevented formation of macroscopic BCC in IR-exposed animals at the age of 16 months [55]. A follow-up study showed that tazarotene also inhibited the number and size of preexisting microscopic BCC lesions [63]. For this purpose, animals were exposed to IR at 2 months of age and the tazarotene treatment was started 5 months later for additional 5 months. Efficacy was also shown for other retinoid-related agents such as ATRA (pan-RAR agonist), AGN195813 (RAR*α* agonist), or AGN194204 (pan-RXR agonist), however, to a lesser extent [63].

In a next study, the antitumoral effects of cyclooxygenases (COX) inhibitors have been analyzed [64]. COX inhibitors belong to nonsteroidal anti-inflammatory drugs, which are thought to prevent the formation of SCC in humans [72]. The COX inhibitors sulindac (nonspecific COX inhibitor), MF-tricyclic (COX2-specific inhibitor) or celecoxib (COX2-specific inhibitor) were administered starting at the age of 6 weeks and BCC were induced 2 weeks later by exposure to UV (3 times/week, continued until the age of 12 months) or IR (once). At the age of 9 months, the burden of microscopic BCC was reduced by 35% in celecoxib-treated animals and by 50–60% in sulindac- or MF-tricyclic-treated mice [64].

In just another study, *Ptch* ^*neo*12/+^ mice were utilized to assess the effect of tea on BCC formation [54]. The rational for this experiment was the observation that green tea may protect against photocarcinogenesis [73]. Green or black tea was added to the drinking water of *Ptch* ^*neo*12/+^ mice beginning from the age of 46 days. UV exposure (3 times/week) was started 2 weeks later. However, neither number nor size of BCC was reduced 5 or 7 months after initial UV exposure [54].

*Pt* *ch* ^*neo*12/+^ mice were also used to analyze the effects of itraconazole, vitamin D3, or CUR61414. Similar to CUR61414, the antifungal compound itraconazole and vitamin D3 derivatives have been shown to inhibit Hh signaling, probably by interaction and inhibition of SMO [61, 66, 74]. In order to accelerate BCC carcinogenesis in these studies, *Ptch* ^*neo*12/+^ mice were crossed to *K14-Cre-ER p53fl/fl* mice and *p53* was deleted in the *Ptch* ^*neo*12/+^ *K14-Cre-ER p53fl/fl *offspring at the age of 6 weeks by injection of 100 *μ*g tamoxifen on 3 consecutive days. Two weeks later, the animals were exposed once to IR. This resulted in visible BCC at the age of 5-6 months. CUR61414 was applied topically twice daily to BCC on the dorsal skin 5 days a week for up to 42 days. This decreased the tumor size by 60%, which was accompanied by inhibition of *Gli1* expression in tumor tissue [65]. The same was shown for itraconazole. When applied orally twice daily for 18 days, itraconazole led to a significant suppression of tumor growth, which was reversible after drug withdrawal [66]. The treatment with vitamin D3 was also effective. Although an impact on tumor size has not been mentioned by the authors, vitamin D3 blocked proliferation and Hh signaling in visible BCC when applied topically 5 days/week for 30 days [67]. 

Altogether, these data show that conventional *Ptch* knockout mice are an extremely valuable tool to analyze the efficacy of new anti-BCC drugs. Still there might be a few drawbacks when using irradiated *Ptch* ^+/−^ animals. First, the onset of tumor formation is variable, with tumors arising at different time points and different sites after exposure to radiation [45]. This may complicate studies, which address the use of drugs in small precursors as opposed to progressed tumors. Second, due to the mode of BCC induction by exposure to IR or UV, the molecular mechanisms responsible for BCC formation are probably very heterogeneous. This heterogeneity may also be reflected by the spectrum of skin tumor, which comprises nodular, superficial as well as infiltrative BCC subtypes, trichoblastomas, and also SCC [45, 53]. These characteristics of irradiated *Ptch* ^+/−^ mice may hamper the evaluation of new treatment strategies designed for targeting specific BCC subtypes. 

### 3.3. Conditional *Ptch* Knockout Mice

With respect to timing of BCC initiation and to investigate defined BCC stages, conditional *Ptch* knockout mice may be a more suitable model. Conditional knockouts also allow for induction of the *Ptch* mutation in specific cell lineages, which is important when seeking for, for example, the identification of the cell of origin of BCC [44, 75].

To our knowledge five different conditional *Ptch *knockout mouse strains have been generated up to date. Of these, only one has been used in a preclinical study targeting BCC [68]. 

#### 3.3.1. Conditional *Ptch* Knockout Mice Targeting Exons 1, 2, or 3 of the *Ptch* Gene

In *Ptch* ^*neo*/*neo*^ mice, exon 3 is flanked by *loxP* sites [76]. The deletion of exon 3 is expected to lead to a premature stop codon and thus to a truncated *Ptch* protein. Indeed, embryos with a homozygous deletion of *Ptch* exon 3 display developmental defects and die at embryonic day 9.5. This is similar to conventional *Ptch* knockouts, in which the homozygous germline mutation results in embryonal lethality between embryonic day 9.0 and 10.5 [51, 52]. In adult *Ptch* ^*neo*/*neo*^ mice, BCC can be induced with tissue-specific Cre drivers. For example, BCC arise in conditional *Krt*6*a* *Cre* *Ptch* ^*neo*/*neo*^ mice after activation of the Krt6a promotor by topical application of retinoic acid (RA) [44]. Expression of Krt6aCre results in a loss of *Ptch* in 40% of interfollicular basal cells and outer root sheath cells of multiple hair follicles. Within 4 weeks, 25% of animals develop basal cell invaginations and after 12 additional weeks 100% of mice suffer from BCC, which show high Hh signaling activity. However, since the Krt6a promotor is also permanently active in the companion cell layer, untreated *Krt*6*a* *Cre* *Ptch* ^*neo*/*neo*^ mice develop epidermal hyperproliferations by 9 to 12 months and suffer from hair loss. These hyperproliferations are associated with hair follicles or sebaceous glands and do not progress to BCC. 

BCC in *Ptch* ^*neo*/*neo*^ mice can also be induced using the skin-specific *K14Cre* or the *MxCre* mouse. The latter strain is transgenic for a Cre recombinase controlled by the interferon-inducible promoter *Mx.1*. Besides heamatopoetic cells, liver, spleen, kidney, lung, gastric epithelium, and other tissues [77], the *Mx.1* promotor is also active in basal cells of the skin [78]. *K*14*Ptch*1^Δ/Δ^ mice (derived from a cross of *K14Cre* and *Ptch* ^*neo*/*neo*^ mice) develop BCC within 3-4 weeks after birth. In *M* *x* *Ptch*1^Δ/Δ^ animals, BCC occur 8–10 weeks after activation of the MxCre by intraperitoneal injection of the immune stimulator polyinosinic-polycytidylic acid (poly(I:C)) on 3 consecutive days. However, due the widespread activity of the *Mx.1 *promotor, activation of MxCre in *Ptch* ^*neo*/*neo*^ mice also ablates *Ptch* in other organs. This result in B- and T-cell defects, thymic atrophy, increased numbers of myeloid progenitors, and loss of osteoblasts [78]. Due to these defects, this model is rather unsuitable for preclinical studies using anti-BCC drugs.

In addition to *Ptch* ^*neo*/*neo*^ mice, other conditional* Ptch* knockout mouse models targeting *Ptch* exons 1, 2, or 3 exist. In the *Ptch*1^*c*/*c*^ mouse model, the *Ptch* exon harboring the first ATG of the *Ptch* gene and exon 2 are flanked by *loxP* sites [79]. According to the provided data and to the precise nomenclature (see [80]), the exon containing the first ATG equates exon 1B. Therefore, the floxed region in the *Ptch*1^*c*/*c*^ mouse model additionally contains the alternative first *Ptch* exons 1 and 1A [80]. After Cre-mediated excision of these exons, the resulting *Ptch*1^Δ*loxP*/Δ*loxP*^ embryos display the same phenotype as homozygous embryos derived from conventional knockouts. However, embryonic and neonatal lethality is also observed in some *Ptch*1^*c*/*c*^ mice, which probably results from *Ptch* misexpression due to the insertion of a *lacZ* gene. 

The *Ptch*1^*c*/*c*^ mouse model is similar to a third *Ptc*1^*F*1-2*m*^ conditional mouse model described by Taniguchi and colleagues, which likewise allows for the ablation of the exons 1B, 1, 1A, and 2 [81]. Finally, one recent publication described *Ptch1neo(fl)Ex2(fl)* mice, which develop BCC-like lesions after activation of the Cre recombinase K5Cre*PR1 by RU486 or of Lgr5-EGFP-IHRES-creER^T2^ by tamoxifen [75]. Although not explicitly mentioned by the authors, we assume that exon 2 is targeted in *Ptch1neo(fl)Ex2(fl)* animals. 

Due to alternative splicing of *Ptch* exons, the above-mentioned animal models may be somewhat leaky when it comes to a complete deletion of all *Ptch* transcript variants. As shown by Shimokawa et al. [80], the first *Ptch* exons 1B, 1 and 1A as well as exons 2–5 can be subjected to alternative splicing. Furthermore, an alternative first exon 1C exists, which is located more than 9 kb upstream of exon 2 and can be spliced into exon 2 or 3 of the *Ptch* transcript [80]. This has also been shown by Nagao et al. [82, 83], who used a different numbering for the alternative first exons and who named the most upstream exon 1A [82, 83]. Although the role of the various *Ptch* splice variants is not completely understood, they are expressed in specific tissues and can modulate Hh signaling to various extents [80, 82, 83]. 

According to Nagao et al., *Ptch* exons 6 to 9 are not subjected to alternative splicing [83]. Therefore, targeting this region is beneficial in order to obtain a complete loss of regular *Ptch* transcripts. 

#### 3.3.2. Conditional *Ptch* Knockout Mice Targeting Exons 8 and 9 of the *Ptch* Gene

We have recently described *Ptch* ^*flox*^ knockout mice (available at http://www.jax.org/: *B6N.129-* *Ptch*1^*tm*1*Hahn*^ */J*, Stock 012457) permitting the conditional ablation of exons 8 and 9 by introduction of *loxP* sites into the introns 7 and 9. *Ptch* ^*flox*/*flox*^ mice are born at the expected Mendelian ratio and are viable and fertile. Neither the *loxP *sites nor the neomycin resistance cassette in intron 9 disturb the normal splicing of the *Ptch* mRNA derived from the *Ptch* ^*flox*^ allele [84]. As reported by our group, the excision of exons 8 and 9 can be carried out very effectively, thereby generating the *Ptch* ^*del*^ allele [20, 84–86]. This results in an aberrant *Ptch* transcript with exon 7 spliced into exon 10 and leads to a frameshift and a premature stop codon. The postulated truncated protein consists of 341 instead of 1093 aminoacids and lacks the sterol sensing domain, the second extracellular loop, and the C-terminus. Due to the lack of appropriate *Ptch* antibodies, we were not able to detect this protein, but the phenotype of *Ptch* ^*del*/*del*^ embryos indicates a complete loss of *Ptch* function. Indeed, all homozygous *Ptch* ^*del*/*del*^ mutants die before embryonic day 10 *in utero*. *Ptch* ^*del*/+^ mice survive and develop malformations at incidences similar to those observed in conventional *Ptch* knockout mice on the same genetic background [20, 85].

**Figure 1:**
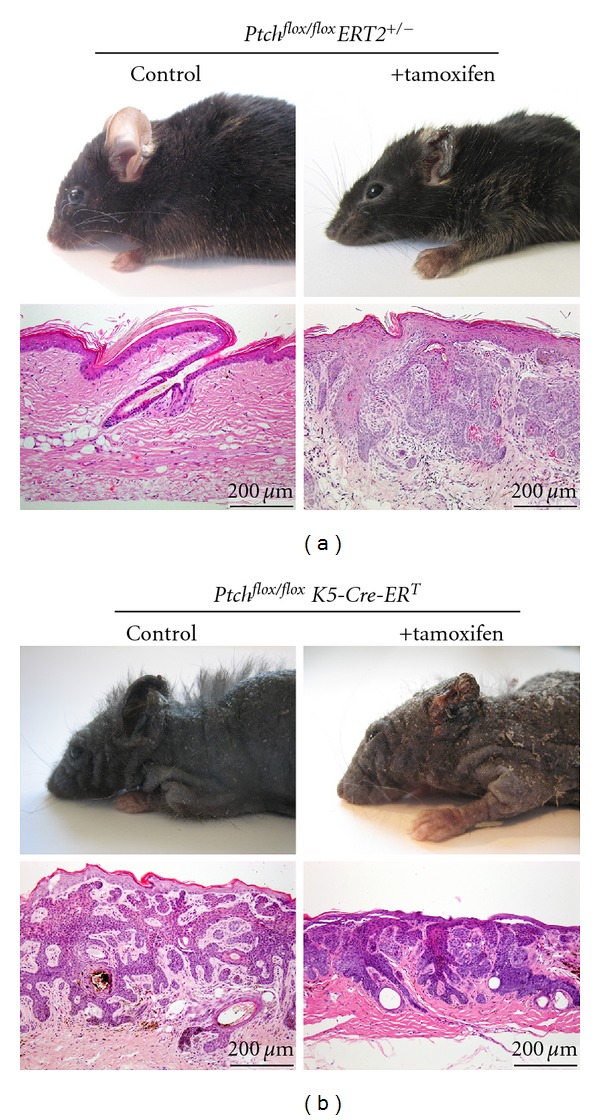
Features of BCC in *Ptch* ^*flox*/*flox*^ *ER* *T*2^+/−^ and *Ptch* ^*flox*/*flox*^ *K*5-*Cre*-*ER* ^*T*^ mice. *Ptch* ^*flox*/*flox*^ animals were bred with the mouse lines *ERT2 *or *K*5-*Cre*-*ER* ^*T*^. The respective *Ptch* ^flox/+^ *ER* *T*2^+/−^ and *Ptch* ^*flox*/+^ *K*5-*Cre*-*ER* ^*T*^ mice were backcrossed to *Ptch* ^*flox*/*flox*^ mice to obtain *Ptch* ^*flox*/*flox*^ *ER* *T*2^+/−^ and *Ptch* ^*flox*/*flox*^ *K*5-*Cre*-*ER* ^*T*^ mice. ERT2 or *K*5-*Cre*-*ER* ^*T*^ was activated by one intramuscular (i.m.) injection of 100 *μ*g tamoxifen as described recently [20, 85], or by intraperitoneal injections of 1 mg tamoxifen (10 *μ*g/*μ*L in sterile ethanol/sun flower oil 1 : 25) for 5 consecutive days (see [88]), respectively. Genotyping of the *Ptch* ^*flox*^, *Ptch* ^*del*^, *ERT2, *and *K*5-*Cre*-*ER* ^*T*^alleles was performed as described recently [20, 84, 85]. All mice used in the study were handled in accordance with the German animal protection law. (a) shows the appearance and histology of skin from control and tamoxifen-treated *Ptch* ^*flox*/*flox*^ *ER* *T*2^+/−^ mice and (b) shows that of control and tamoxifen-treated *Ptch* ^*flox*/*flox*^ *K*5-*Cre*-*ER* ^*T*^ mice.

For the induction of BCC, *Ptch* ^*flox*/*flox*^ mice can be crossed to *Rosa26CreERT2* mice (hereafter *ERT2* mice) that express a tamoxifen-inducible Cre recombinase under the control of the ubiquitously active *ROSA26* promoter [87]. Activation of ERT2 by a single intramuscular (i.m.) injection of 100 *μ*g tamoxifen results in BCC in 100% of animals. Microscopically, BCC precursors are visible 45 days after tamoxifen induction. The tumors are fully developed by day 90 (Figure 1(a)) [85]. After that time, the tumors start to regress, which is becoming obvious 200 days after tamoxifen-treatment [20]. All BCC in this model have features of the human nodular subtype and are noninvasive. As indicated by abundant *Gli1* and *Ptch* expression, they are characterized by strong Hh signaling activity [20, 85]. The tumors develop preferentially on ears and tails and are very rarely detected in hairy skin. The reason for this preference is unknown but may involve a better blood circulation in ears and tails, resulting in elevated tamoxifen concentrations after i.m. application (discussed in [85]). 

This shows that in *Ptch* ^*flox*/*flox*^ *ER* *T*2^+/−^ mice BCC can be induced very easily and reliably by one single injection of tamoxifen. Furthermore, in this model, where all mice show full developed BCC 90 days after activation of ERT2, specific antitumor treatments can be commenced at specific time points after tumor induction and at a defined age of the animals.

As indicated in Table 1, we recently examined the antitumor effects of calcitriol, which is the physiologically active form of vitamin D3 [68]. Calcitriol treatment was started either immediately or 60 days after BCC initiation. The treatment was continued until day 90, when all mice were sacrificed. The study revealed that BCC growth was significantly inhibited in mice treated from days 0 to 90, but not in those treated from days 60 to 90 [68]. These data show that conditional *Ptch* ^*flox*/*flox*^ *ER* *T*2^+/−^ mice are particularly useful to study the preventive or curative effects of a specific anticancer drug. This is due to the reliable BCC initiation and progression to early precursors (after 45 days) and fully developed (after 90 days) tumors. 

**Figure 2:**
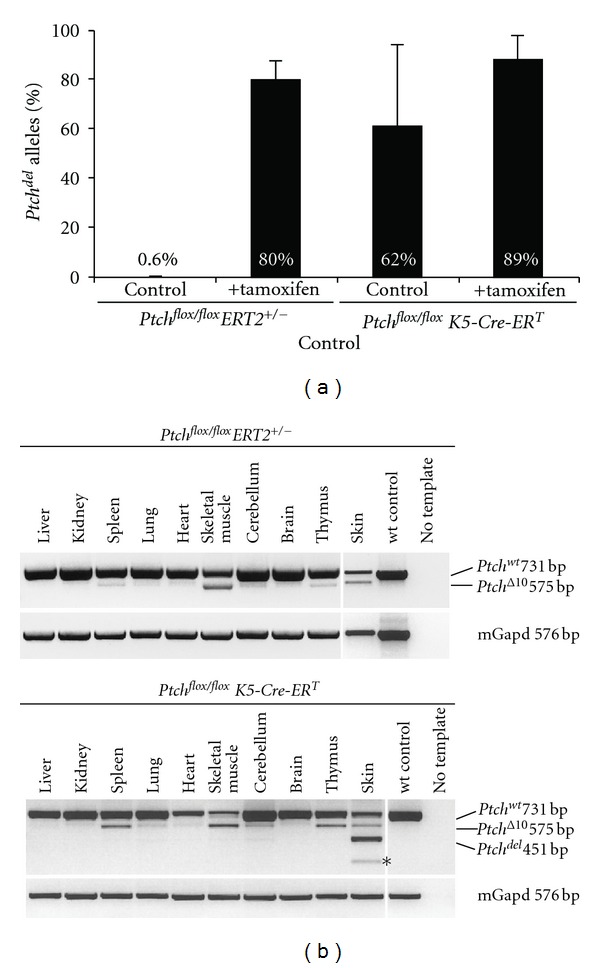
*Ptch* recombination and expression in *Ptch* ^*flox*/*flox*^ *ER* *T*2^+/−^ and *Ptch* ^*flox*/*flox*^ *K*5-*Cre*-*ER* ^*T*^ mice. (a) The efficiency of* loxP* recombination at the *Ptch* locus in DNA derived from tail skin from untreated and tamoxifen treated mice was determined by allele-specific real-time PCR as described in [85]. (b) *Ptch* transcripts in different tissues were analyzed by RT-PCR. The transcripts derived from the *Ptch* ^*flox*^ and the *Ptch* ^*del*^ locus (the latter equates to the floxed *Ptch* locus after Cre-mediated excision) were analyzed by semiquantitative RT-PCR as described in [85]. In the skin, untreated *Ptch* ^*flox*/*flox*^ *ER* *T*2^+/−^ mice only expressed *Ptch* ^*wt*^ transcripts and the normally occurring *Ptch* ^Δ10^ transcript lacking exon 10. In contrast, untreated *Ptch* ^*flox*/*flox*^ *K*5-*Cre*-*ER* ^*T*^ mice expressed *Ptch* ^*del*^ transcripts and *Ptch* ^*del*^ transcripts lacking exon 10 (asterisk) in the skin.

Although the *Ptch* ^*flox*/*flox*^ *ER* *T*2^+/−^ BCC model is very easy to handle (i.e., application of one single dose of tamoxifen) and is solid with respect to induction of a specific BCC subtype (i.e., 100% of animals develop the nodular subtype 90 days after BCC induction), it also may have some disadvantages. Due to the ubiquitous expression of *ERT2*, the i.m. application of even a low dose of tamoxifen may cause *Ptch* deletion in other cell lineages or tissues. Even though we have not found any evidence for *Ptch*-ablation in other organs than the epidermis and the injected muscle [20, 85], we now have crossed *Ptch* ^*flox*/*flox*^ animals to *K*5-*Cre*-*ER* ^*T*^ mice, which express the tamoxifen-inducible *ER* ^*T*^ selectively in cells of the basal layer of the skin [89–92]. Activation of *ER* ^*T*^ by 4-hydroxy-tamoxifen (the active metabolite of tamoxifen) is ~10-fold less efficient than that of ERT2 [89]. Therefore, a cumulative dose of 5 mg has been used to activate *K*5-*Cre*-*ER* ^*T*^ in 10-weeks-old *Ptch* ^*flox*/*flox*^ *K*5-*Cre*-*ER* ^*T*^ animals. Untreated *Ptch* ^*flox*/*flox*^ *K*5-*Cre*-*ER* ^*T*^ mice served as controls. All *Ptch* ^*flox*/*flox*^ *K*5-*Cre*-*ER* ^*T*^ mice developed BCC on tails and ears after tamoxifen injection (Figure 1(b)). They also suffered from BCC in hairy skin. Importantly, BCC also developed in *Ptch* ^*flox*/*flox*^ *K*5-*Cre*-*ER* ^*T*^ untreated control mice. Some control mice even suffered from complete hair loss (Figure 1(b)). Histological examination revealed that 86% of the controls (12/14) have developed BCC at the age of 87–172 days (Table 2). After 200–246 days, all control mice have developed tumors even without *K*5-*Cre*-*ER* ^*T*^ activation (Table 2, Figure 1(b)). The leakiness of *K*5-*Cre*-*ER* ^*T*^ was also demonstrated on molecular level. Thus, the amount of recombined *Ptch* alleles in DNA isolated from skin derived from untreated controls was up to 83% (*n* = 5, mean 62%). This was almost identical to tamoxifen-treated *Ptch* ^*flox*/*flox*^ *K*5-*Cre*-*ER* ^*T*^ animals (amount of recombined *Ptch* allels 91%, *n* = 6) (Figure 2(a)). Consistent with these data, untreated *Ptch* ^*flox*/*flox*^ *K*5-*Cre*-*ER* ^*T*^ mice also showed high expression of *Ptch* ^*del*^ transcripts in the skin (Figure 2(b)).

This is considerably different to untreated *Ptch* ^*flox*/*flox*^ *ER* *T*2^+/−^ mice, which do not develop any skin tumors within up to 293 days (*n* = 13) (Table 2), and which do not show recombination at the floxed *Ptch* locus in the absence of tamoxifen. Thus, whereas the recombination is 89% in tamoxifen-treated mice (*n* = 4), it is only 0.6% in untreated *Ptch* ^*flox*/*flox*^ *ER* *T*2^+/−^ animals (*n* = 7) and *Ptch* ^*del*^ transcripts were never detected in any of the examined tissues (Figure 2(a)).

Although BCC in both the *Ptch* ^*flox*/*flox*^ *ER* *T*2^+/−^ and *Ptch* ^*flox*/*flox*^ *K*5-*Cre*-*ER* ^*T*^ model are identical based on histology and also at the level of Hh signaling activity (i.e., BCC of both models express *Gli1* and *Ptch*), *K*5-*Cre*-*ER* ^*T*^ is highly leaky resulting in BCC formation even without *Cre* activation. Therefore, *K*5-*Cre*-*ER* ^*T*^ should not be used in combination with *Ptch* ^*flox*/*flox*^ mice if exact timing of BCC induction is of interest. However, since leakiness of *CreER* lines can differ between conditional mouse strains [93] it remains to be elucidated whether *K*5-*Cre*-*ER* ^*T*^ leakiness is also seen in other conditional *Ptch* models.

## 4. Conclusion

BCC is the most common cancer in humans. Due to their high and increasing incidence, the improvement of current treatment options and the development of new treatment approaches are of great importance. Based on the essential role of HH signaling in formation of BCC, targeting this pathway is currently being put forward (for a review on 36 HH inhibitory compounds see [94]). The preclinical evaluation of these anti-BCC drugs requires good animal models. General requirements for such a model are a close relationship to the human situation (i.e., BCC caused by *Ptch* mutations on an immunocompetent background), reliable induction of BCC, defined BCC growth, and easy handling.

We have compared several *Ptch* knockout mouse models suitable for preclinical studies. So far, most studies have been conducted in UV- or IR-exposed conventional heterozygous *Ptch* knockout mice. Whereas UV-exposure leads to both superficial and nodular BCC and several other tumors, IR-exposure results in nodular and infiltrative BCC. Although the UV- or IR-related BCC models are valuable tools to analyze the antitumoral response of BCC, the responsiveness of defined BCC stages (i.e., early-stage or fully developed) or subtypes (e.g., nodular or superficial) in these models is hard to analyze due to heterogeneous BCC growth. For this purpose, conditional inactivation of *Ptch* by inducible and cell-specific Cre drivers may be advantageous. Five different conditional* Ptch* knockout mouse strains are currently available. However, preclinical studies on anti-BCC drugs have only been carried out in one of them. As revealed by this study, conditional *Ptch* ablation indeed enables the investigator to accurately induce BCC at a defined time point. In addition, conditional *Ptch* ablation results in a homogeneous BCC histology, which may be due to omission of irradiation. Therefore, conditional *Ptch* knockout mice are a valuable tool to study the curative or preventive effects of a certain drug on defined BCC subtypes and stages.

## Figures and Tables

**Table 1:** *Ptch* knockout mouse models for preclinical BCC treatment studies.

BCC model	Mode of BCC induction	Treatment	Reference
*Pt* *ch* ^*neo*12/+^	UV	Cyclopamine	[60]
*Pt* *ch* ^*neo*12/+^ skin punches	UV	CUR61414	[61]
*Pt* *ch* ^*neo*12/+^	UV	*α*-difluoromethylornithine	[62]
*Pt* *ch* ^*neo*12/+^	IR/UV	Tazarotene	[55]
*Pt* *ch* ^*neo*12/+^	UV	Tazarotene, ATRA, AGN195813, AGN194204, AGN194310	[63]
*Pt* *ch* ^*neo*12/+^	IR/UV	Celecoxib, sulindac, MF-tricyclic	[64]
*Pt* *ch* ^*neo*12/+^	UV	Green/black tea	[54]
*Pt* *ch* ^*neo*12/+^ * K14-Cre-ER p53fl/fl*	IR and conditional p53 ablation	CUR61414	[65]
*Pt* *ch* ^*neo*12/+^ * K14-Cre-ER p53fl/fl*	IR and conditional p53 ablation	Itraconazole	[66]
*Pt* *ch* ^*neo*12/+^ * K14-Cre-ER p53fl/fl*	IR and conditional p53 ablation	Vitamin D3	[67]
*Pt* *ch* ^*flox*/*flox*^ *ER* *T*2^+/−^	conditional *Ptch* ablation	Calcitriol	[68]

UV: ultraviolet radiation; IR: ionizing radiation.

**Table 2:** BCC formation in *Ptch* ^*flox*/*flox* *flox*^ *ER* *T*2^+/−^ and *Ptch* ^*flox*/*flox*^ *K*5*Cre* *ER* *T* ^+/−^ mice.

Genotype	Age at tamoxifen application	*n*	Age range (days)	Mice with BCC	Healthy
*Pt* *ch* ^*flox*/*flox* *flox*^ *ER* *T*2^+/−^	—	13	56–293	0	13
*Ptch ^*flox*/*flox* *flox*^ *ER* *T*2^+/−^	42–56 days	10	93–365	10	0
*Pt* *ch* ^*flox*/*flox*^ *K*5*Cre* *ER* *T* ^+/−^	—	14	87–172	12	2
*Pt* *ch* ^*flox*/*flox*^ *K*5*Cre* *ER* *T* ^+/−^	—	14	200–246	14	0
*Pt* *ch* ^*flox*/*flox*^ *K*5*Cre* *ER* *T* ^+/−^	55–82 days	9	132–170	9	0

*Data already published in [85].

## Abbreviations

Ptch: Patched
BCC: Basal cell carcinoma.
